# A Fatal Herpes Simplex Encephalitis with a Normocellular Cerebrospinal Fluid: A Case Report

**DOI:** 10.3390/jcm15176704

**Published:** 2026-08-29

**Authors:** Karolina Dutkowska, Krystian Ejdys, Marcin P. Mycko

**Affiliations:** Department of Neurology and Neurosurgery, University of Warmia and Mazury in Olsztyn, Warszawska 30, 10-082 Olsztyn, Poland; karolina.dutkowska-kuzba@uwm.edu.pl (K.D.); marcin.mycko@uwm.edu.pl (M.P.M.)

**Keywords:** herpes simplex virus type 1, encephalitis, cerebrospinal fluid, case report

## Abstract

**Background:** Herpes simplex encephalitis (HSE) is a rare but life-threatening infection of the central nervous system associated with high mortality and severe neurological sequelae. The diagnosis may be particularly challenging in patients presenting with atypical clinical or laboratory findings. **Case presentation:** An 85-year-old woman presented with progressive impairment of consciousness and generalized neurological deterioration. Initial neuroimaging suggested an acute ischemic stroke, whereas subsequent brain magnetic resonance imaging (MRI) demonstrated bilateral temporal and insular cortical abnormalities suggestive of encephalitis. Cerebrospinal fluid (CSF) analysis revealed no pleocytosis, and blood tests showed no markers of systemic inflammation during admission, which initially lowered the suspicion of infectious encephalitis. Although no inflammatory cells were detected in the routine CSF examination, polymerase chain reaction (PCR) testing confirmed infection with herpes simplex virus type 1 (HSV-1). Despite the prompt initiation of acyclovir after suspicion of encephalitis with MRI, together with corticosteroid therapy, anticonvulsants, and supportive treatment, the clinical course was fatal. **Conclusions:** This case highlights the diagnostic challenges associated with HSE, particularly in patients with normocellular CSF and radiological findings that may mimic other neurological conditions, including ischemic stroke. The absence of CSF pleocytosis does not exclude HSE, although the timing of CSF sampling should be considered when interpreting this finding. Early MRI and molecular CSF diagnostics remain essential for establishing an accurate diagnosis and ensuring timely treatment.

## 1. Introduction

Herpes simplex virus encephalitis (HSE) is one of the most severe viral infections of the central nervous system (CNS) and is associated with a high risk of mortality and significant long-term neurological sequelae among survivors [1]. It is a rare neurological disorder characterized by inflammation of the brain parenchyma, with a particular predilection for the mesial temporal lobes. Approximately 90% of cases are caused by herpes simplex virus type 1 (HSV-1), while herpes simplex virus type 2 (HSV-2) accounts for fewer than 10% of cases [1,2].

Swedish researchers found that the incidence of HSE across all age groups—from infants under one year to individuals over 90 years of age—was 2.2 cases per million people annually [3]. A nationwide, prospective, population-based cohort study using the Danish Brain Infection Study Group database was conducted to identify all adults hospitalized with microbiologically confirmed HSV-1 encephalitis in Denmark between 2015 and 2023. A total of 154 patients with confirmed HSV-1 encephalitis were included, corresponding to a mean annual incidence of 0.36 cases per 100,000 population [4]. The clinical presentation of HSE is heterogeneous and includes nonspecific symptoms such as fever, headache, nausea, focal neurological deficits, altered mental status, seizures, and behavioral changes [2,5]. The disease can occur at any age; however, it is most common and often more severe in children and the elderly [1,2,6,7]. Due to the nonspecific nature of early symptoms, diagnosis may be challenging and requires appropriate diagnostic evaluation, particularly cerebrospinal fluid (CSF) analysis and neuroimaging studies [1,6,8,9,10]. Cerebrospinal fluid (CSF) analysis in patients with HSE typically demonstrates lymphocytic pleocytosis. CSF white blood cell counts generally range from 5 to 500 cells/mm^3^, with lymphocytes accounting for approximately 60–98% of the cellular population. Additionally, a normal or mildly elevated opening pressure, elevated protein concentration, and normal glucose levels in the CSF may be present. Although these findings are not specific to HSE, they are highly suggestive of viral encephalitis and constitute an important component of the diagnostic evaluation [1,6].

We present the case of an elderly woman who was ultimately diagnosed with HSE, highlighting the diagnostic complexity and atypical clinical presentation of this condition. Through this analysis, we aim to highlight diagnostic challenges and the possibility of normal routine CSF findings despite severe ongoing CNS inflammation.

## 2. Case Presentation

An 85-year-old woman presented to the Department of Neurology, University Hospital in Olsztyn, Poland, on 20 March 2026, due to impaired consciousness. According to the history obtained from the patient’s son, the patient had been experiencing progressive drowsiness, reduced responsiveness, and fever up to 38 °C for five days prior to admission. The son also reported that the patient had shingles in the back area in December 2025. On 15 March 2026, she was evaluated in the emergency department of a regional hospital, where a non-contrast head computed tomography (CT) scan was performed, reportedly demonstrating only the presence of chronic ischemic changes, and the patient was discharged home the following day (16 March 2026). Six days preceding neurological hospitalization, the patient had significantly reduced oral intake, consuming only fluids and no solid meals. Her past medical history was notable for Alzheimer’s-type dementia. She was not receiving any regular medication. There was no available information regarding her allergies.

The patient was admitted in a severe general condition. On admission, the patient was cachectic, dehydrated, and presented with dry skin. Laboratory findings revealed no evidence of systemic inflammation or infection, with normal blood CRP and procalcitonin levels and no leukocytosis. The patient was conscious but did not establish logical verbal contact and was unable to follow commands. Responses to painful stimuli were markedly reduced. Oxygen therapy was initiated using a reservoir mask at a flow rate of 10 L/min, resulting in an oxygen saturation of 90%. Blood pressure was 117/58 mmHg, and heart rate was 70 beats per minute. Neurological examination demonstrated generalized increased muscle tension without evident meningeal signs, including a lack of neck stiffness. Joint contractures were noted. The pupils were bilaterally narrow, symmetrical, and reactive to light. No nystagmus was observed, and cranial nerve function appeared preserved. Generalized muscle weakness involving all four limbs was present, with increased muscle tone. Tendon reflexes were preserved and symmetrical, but both ankle jerks were absent. Bilateral plantar responses were flexor. Assessment of sensory modalities and coordination was not possible due to the patient’s clinical condition. The patient was immobile. Given the pre-existing Alzheimer’s-type dementia and markedly impaired level of consciousness, a reliable assessment of memory, higher cortical functions, and cognitive changes from baseline could not be performed.

**Figure 1:**
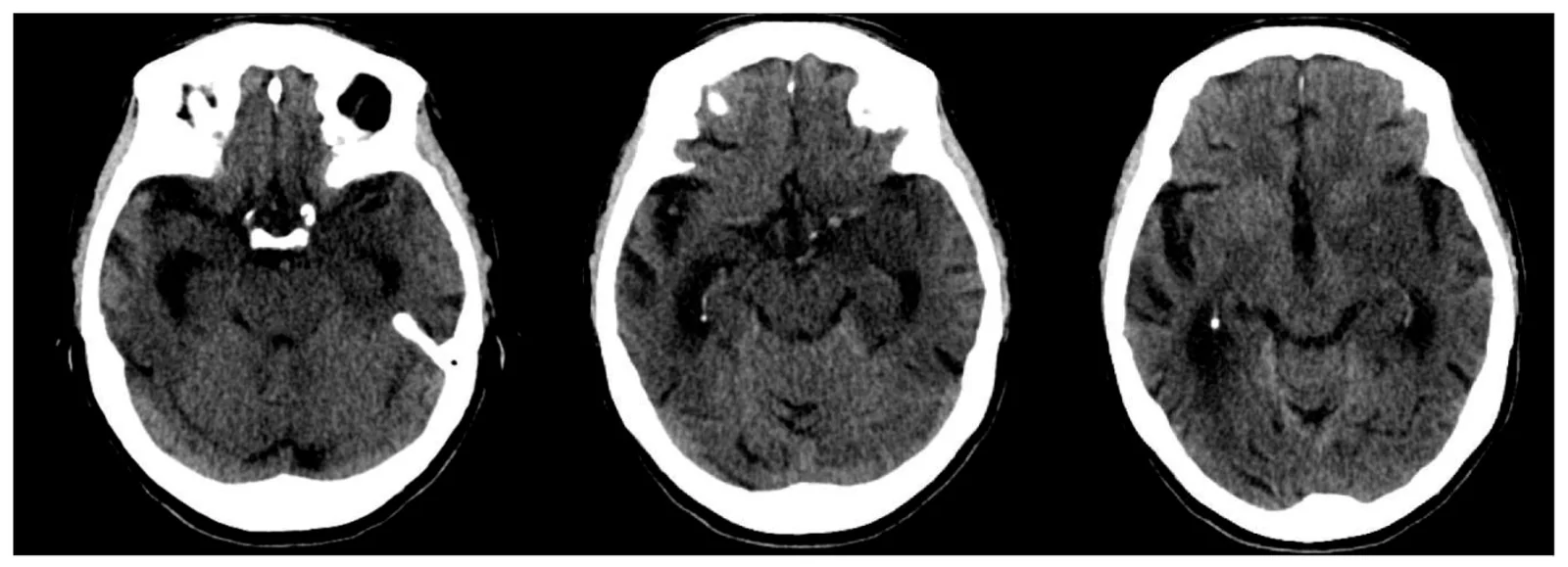
Head axial CT performed on 20 March 2026. A hypodense lesion in the left insular cortex with poorly defined margins, suggestive of a potential acute ischemic stroke, was observed.

Acute non-contrast head CT demonstrated a hypodense lesion in the left insular cortex with poorly defined margins, suggestive of an acute ischemic stroke. No evidence of intracranial hemorrhage was found. Gray-white matter differentiation was preserved. Hypodense changes in the periventricular white matter were observed, consistent with leukoaraiosis. Additionally, features of cortical–subcortical atrophy were present (Figure 1).

Laboratory investigations revealed hyponatremia (Na: 125 mmol/L), along with borderline hypokalemia (K: 3.5 mmol/L). Urinalysis indicated the presence of bacteria, suggestive of a urinary tract infection. No evidence of systemic inflammation was observed, with normal CRP and procalcitonin levels and no leukocytosis. The patient was treated with antiplatelet agents, low-molecular-weight heparin, empirical antibiotics, intravenous fluids, and electrolyte supplementation. Rehabilitation was also initiated.

**Figure 2:**
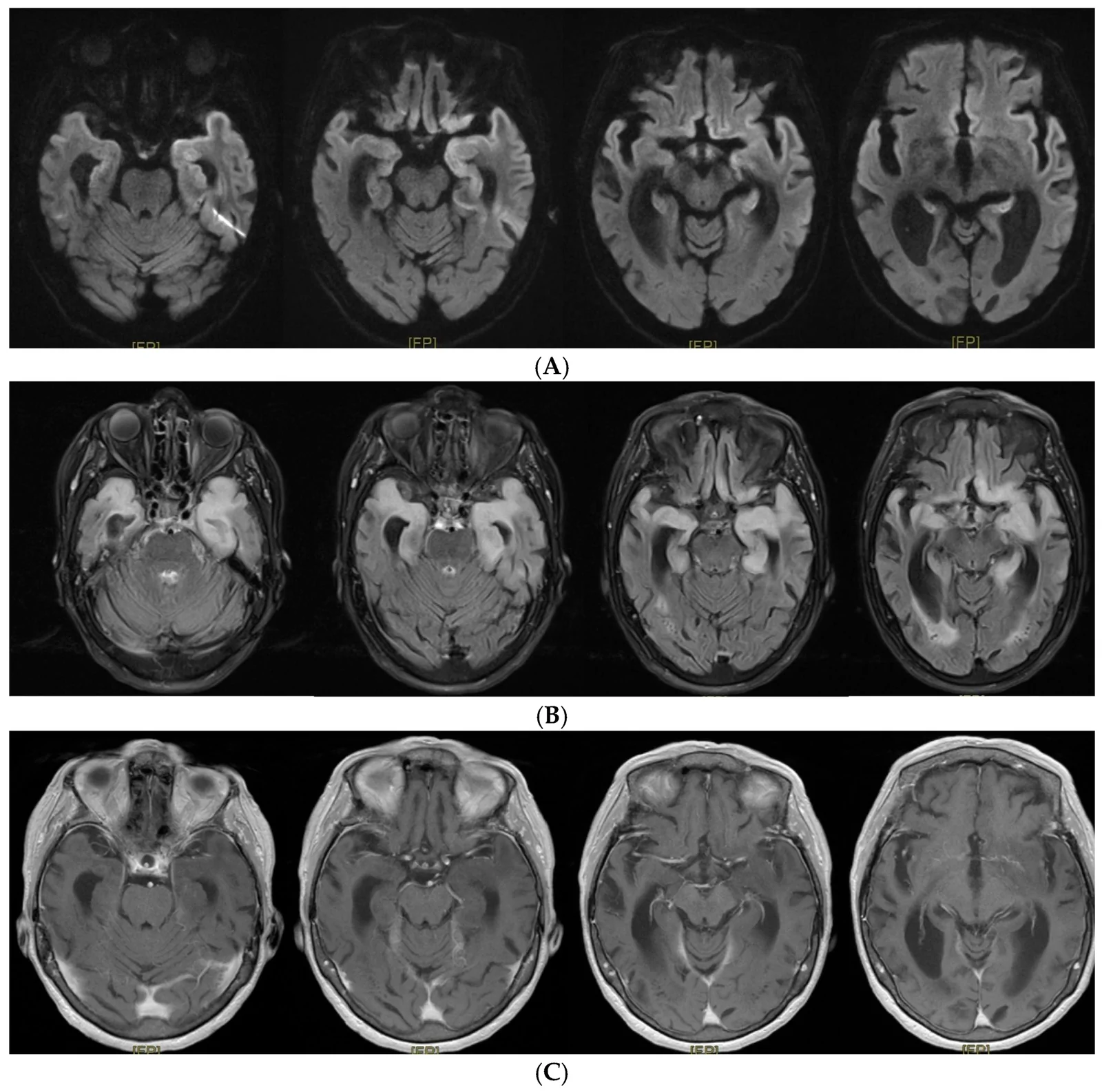
Magnetic resonance imaging (MRI) of the patient: (**A**) Diffusion-weighted imaging (DWI) MRI images. Bilateral cortical diffusion restriction involving the temporal lobes, insular cortices, and basal regions of the frontal lobes, with additional involvement of the mesial temporal structures, including the hippocampi and amygdala, as well as the anterior cingulate gyri, with a clear predominance on the left side. (**B**) Fluid-attenuated inversion recovery (FLAIR) MRI images. Hyperintense lesions involving both temporal lobes and the insular cortices, with involvement of the mesial temporal structures, hippocampi, amygdala, orbitofrontal cortex, and anterior cingulate gyri, more pronounced on the left side. (**C**) Post-contrast T1-weighted MRI images. No pathological contrast enhancement was observed following intravenous administration of a gadolinium-based contrast agent.

As the patient’s condition did not improve, magnetic resonance imaging (MRI) of the brain was performed. MRI revealed abnormalities involving the mesial temporal structures, including the hippocampi and amygdala, with a clear predominance in the left hemisphere. Abnormal signal changes were also observed in the orbitofrontal cortex bilaterally, more pronounced on the left side, as well as in the anterior portions of the cingulate gyri in both cerebral hemispheres. These findings were accompanied by nonspecific signal abnormalities on conventional sequences. Numerous small supratentorial foci of hemosiderin deposition were also noted, consistent with chronic microbleeds. No pathological contrast enhancement was observed following intravenous administration of a gadolinium-based contrast agent. The corpus callosum appeared normal in width and signal intensity. Additionally, imaging demonstrated features of acute inflammatory changes in the right maxillary sinus and right mastoid air cells (Figure 2). The overall radiological findings of MRI were suggestive of encephalitis, particularly involving the limbic system.

Approximately 1–2 h after the MRI, an urgent lumbar puncture was performed. At this time point, at least 8 days after the onset of the febrile illness, CSF analysis revealed no pleocytosis (cytosis 0), an elevated protein level (113 mg/dL), and an elevated glucose level (89.40 mg/dL). CSF chloride level was within reference values (122.90 mmol/L).

Based on the MRI findings, the patient received intravenous acyclovir at a dose of 10 mg/kg every 8 h. Given the atypical presentation and absence of CSF pleocytosis, autoimmune encephalitis was also suspected, and intravenous methylprednisolone pulse therapy (1 g/day) was initiated, together with proton pump inhibitors and analgesics.

**Figure 3:**
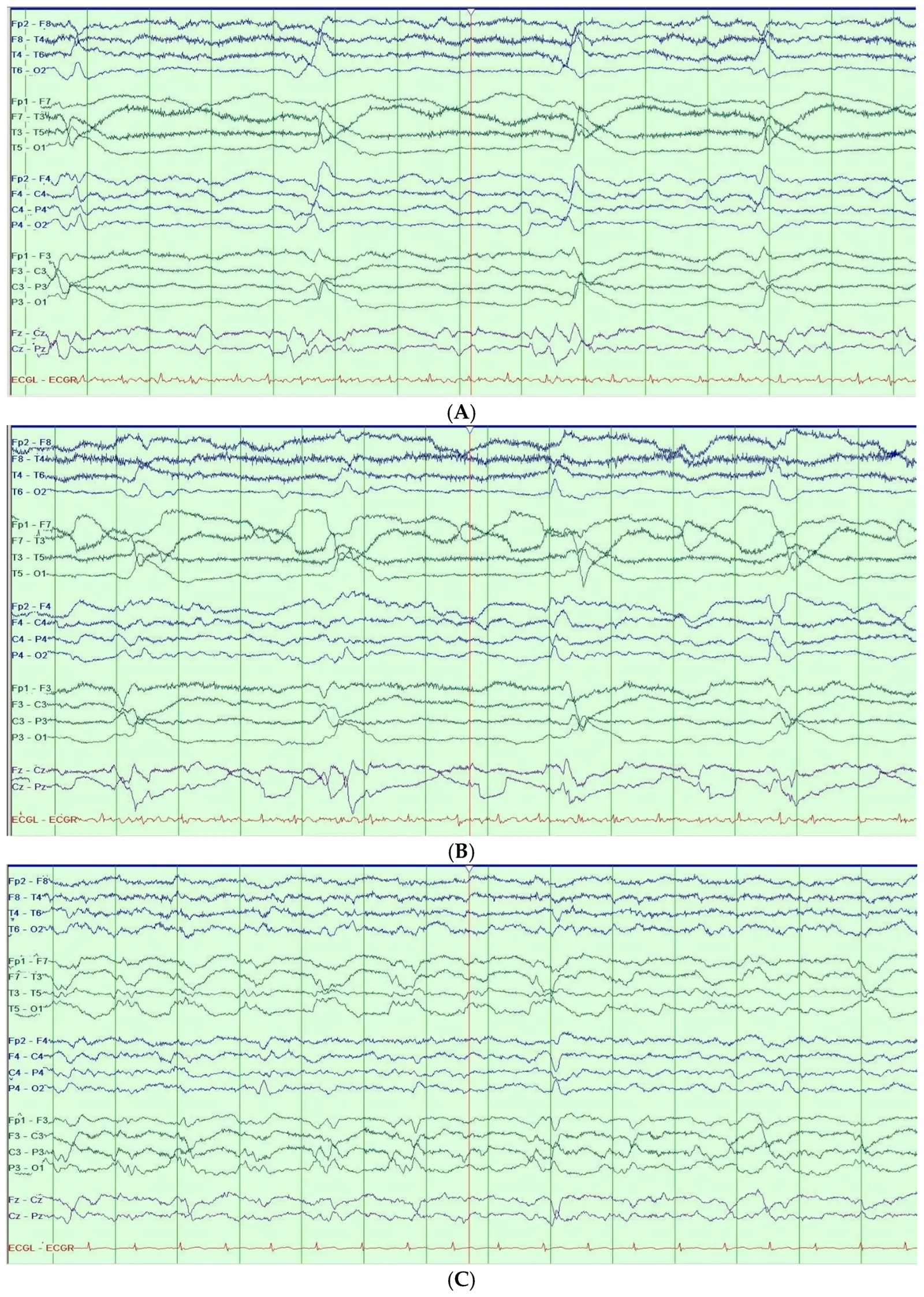
Patient’s EEG recordings: (**A**,**B**) EEG performed on 23 March 2026. Bilateral epileptiform activity. (**C**) Follow-up EEG performed on 24 March 2026. Epileptiform discharges in the left cerebral hemisphere, characterized by sharp waves and theta activity at a frequency of 6–7 Hz, with amplitudes reaching 80–120 µV.

The first video electroencephalography (video-EEG) revealed paroxysmal discharges of sharp waves and theta waves of 5–6 Hz with an amplitude of up to 130–150 µV, predominating in the left hemisphere of the brain, with independent but also synchronized discharges of a similar nature in the right hemisphere of the brain with a frequency of once every 3–4 s and of a non-rhythmic periodic nature. Anticonvulsant therapy was initiated (valproic acid) (Figure 3A,B). Follow-up EEG the following day demonstrated epileptiform discharges in the left cerebral hemisphere, characterized by sharp waves and theta activity at a frequency of 6–7 Hz, with amplitudes reaching 80–120 µV. These discharges showed a tendency toward secondary generalization and were less frequent compared to the prior examination (Figure 3C).

Abdominal CT was performed, which showed no signs of cancer or ovarian teratoma. Two days after lumbar puncture, the results of a multiplex CSF PCR for 14 pathogens were obtained. The panel included CMV, EV, HSV-1, HSV-2, HHV-6, HPeV, VZV, Escherichia coli, Haemophilus influenzae, Listeria monocytogenes, Neisseria meningitidis, Streptococcus agalactiae, Streptococcus pneumoniae, and Cryptococcus neoformans/gattii. Polymerase chain reaction (PCR) testing of the CSF was positive for HSV-1 DNA, while all other results were negative. Serological testing of blood for autoimmune encephalitis antibodies, including anti-AMPA, GABA-B, NMDA, DPPX, CASPR2, and LGI1 IgG, was negative. Testing for Lyme disease and tick-borne encephalitis was also negative, as were blood tests for anti-onconeural antibodies.

Despite the continued treatment, the patient remained in a severe condition, nonverbal, requiring oxygen therapy via a reservoir mask, and was maintained on enteral nutrition with a commercially prepared enteral formula. On the fifth day of hospitalization, the patient developed pneumonia that led to respiratory failure. Metabolic disturbances were observed, including transient hyponatremia and transient hypokalemia, which were corrected with treatment. The patient’s condition remained critical. Ongoing management included fluid therapy with electrolyte correction, antibiotic and antiviral treatment, corticosteroid therapy, enteral nutrition, and continuous analgesia with a morphine infusion. Clinical features of respiratory failure were observed, including tachypnea, oxygen desaturation to 80% despite passive oxygen therapy via a reservoir mask at a flow rate of 10–12 L/min, shallow respirations, and an abdominal breathing pattern. On the sixth day of hospitalization, despite continued valproate treatment, the patient developed a generalized seizure, followed by tachycardia, circulatory centralization, and a further decline in oxygen saturation to 50%. The patient became unresponsive, with a Glasgow Coma Scale score of 3. The airways were cleared, and oxygen therapy via a reservoir mask was continued. Due to the patient’s advanced age, severe frailty, pre-existing Alzheimer’s-type dementia, profound neurological impairment, progressive respiratory failure, and extremely poor overall prognosis, the anesthesiology team classified the patient as priority 4 for intensive care admission. In accordance with the clinical assessment and the established treatment plan, advanced medical interventions, including endotracheal intubation and invasive mechanical ventilation, were not pursued, and supportive treatment was continued. The patient subsequently developed respiratory and circulatory arrest. On 26 March 2026, the patient was pronounced dead.

**Figure 4:**
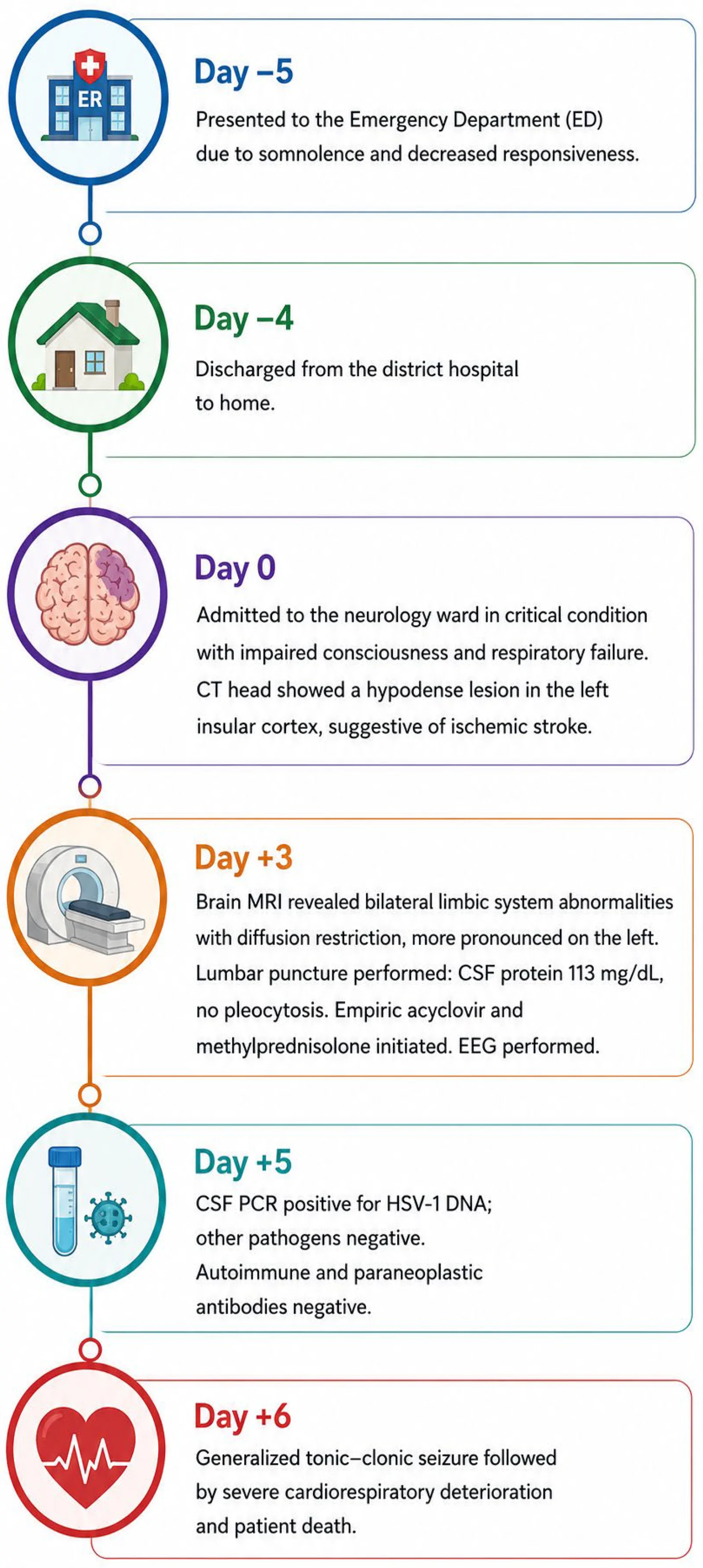
Clinico-diagnostic timeline of the clinical course of HSE.

Initially, acute ischemic stroke and autoimmune encephalitis were considered due to the early neuroimaging findings, while the absence of CSF pleocytosis and systemic inflammatory markers made the diagnosis of infectious encephalitis challenging. Finally, based on the clinical presentation, cumulative imaging findings, and results of additional tests, the patient was diagnosed with HSE. Therefore, no autopsy was performed. The detailed clinico-diagnostic timeline of HSE development in this patient is illustrated in Figure 4.

## 3. Discussion

This case illustrates a complex diagnostic pathway in HSE, in which an initial CT finding suggestive of acute ischemic stroke, absence of CSF pleocytosis, limited neurological assessment, and subsequent characteristic MRI and EEG abnormalities collectively contributed to diagnostic uncertainty. According to the consensus statement of the International Encephalitis Consortium on case definitions, diagnostic algorithms, priorities in encephalitis, and criteria for the diagnosis of encephalitis and encephalopathy of suspected infectious or autoimmune origin, a CSF WBC count ≥ 5/mm^3^ is considered a minor criterion. Furthermore, the absence of CSF pleocytosis does not exclude encephalitis [11]. With regard to HSE, only several published cases have documented the absence of CSF pleocytosis. HSE without cytosis was described in a 72-year-old man treated at The Hague Teaching Hospital in the Netherlands [12]. Despite two lumbar punctures performed, cytosis in cerebrospinal fluid was within the reference range. PCR of cerebrospinal fluid obtained during the second lumbar puncture was positive for HSV type 1; subsequent analysis of the sample from the first lumbar puncture also confirmed HSV-1 positivity. In this case, hypodense changes were also observed in the head CT within the temporal lobe; however, they were not described as acute ischemic stroke but as edematous changes. Despite treatment, the patient ultimately died [12]. The suspicion of autoimmune encephalitis and administration of intravenous methylprednisolone were also described in a case report of a 63-year-old man who presented with his first-ever seizure [13]. Fever did not appear until the fourth day of hospitalization, and initial neuroimaging and CSF analysis showed no abnormalities. However, once the patient developed a fever, repeat neuroimaging was performed, revealing findings consistent with HSE, and PCR tests for HSV type 1 in the serum and CSF were positive. After treatment, the patient was discharged without any sequelae [13]. Two patients with PCR-confirmed HSE despite the absence of CSF pleocytosis were reported in France [14]. The first patient, a 90-year-old man, presented with a fever (39.9 °C), confusion, and no focal neurological signs. Brain CT was unremarkable. CSF examination disclosed 1 leukocyte/μL (normal <5 leukocytes/μL) and a protein level of 1.23 g/L (normal <0.40 g/L), but HSV-1 in the CSF was positive according to PCR. Following treatment and clinical improvement, the patient was discharged home. The second case involved an 80-year-old man who was referred due to dyspnea. On the day following admission, he developed a generalized tonic–clonic seizure accompanied by a fever of 39 °C, without focal neurological deficits. Brain CT was unremarkable, while electroencephalography showed frontotemporal slowing. Cerebrospinal fluid analysis revealed 1 leukocyte/μL and a protein level of 0.78 g/L. HSV-1 PCR in the CSF was positive. Despite temporary clinical improvement, the patient’s neurological condition subsequently deteriorated, progressing to partial status epilepticus, and he died five days after admission [14].

HSE can mimic several acute neurological conditions, including an acute ischemic stroke. In our patient, the initial CT finding of a hypodense lesion in the left insular cortex was considered suggestive of acute ischemic stroke. However, this finding alone was insufficient to explain the overall clinical presentation, particularly the combination of fever, progressive impairment of consciousness, and generalized neurological deterioration. The subsequent MRI demonstrated bilateral temporal and insular cortical abnormalities, with additional involvement of the mesial temporal structures, including the hippocampi and amygdala, as well as the orbitofrontal cortex and anterior portions of the cingulate gyri bilaterally, with a clear predominance of abnormalities in the left hemisphere. This distribution of abnormalities supported an encephalitic process rather than an isolated ischemic lesion. This case also emphasizes the potential value of early brain MRI in patients with impaired consciousness when the clinical history and neurological examination are limited [15]. In such circumstances, MRI may reveal characteristic temporal and insular abnormalities that are not sufficiently specific or may be misleading on initial CT imaging, thereby supporting earlier consideration of encephalitis and prompt CSF molecular testing. In the present case, antiviral therapy with acyclovir was initiated promptly once MRI findings raised suspicion of encephalitis. However, treatment was commenced only after several days of clinical deterioration, reflecting the diagnostic challenge posed by the atypical presentation and the absence of characteristic inflammatory findings in the cerebrospinal fluid. This prolonged interval between symptom onset and initiation of antiviral therapy may have contributed to the unfavorable outcome and highlights the importance of maintaining a high clinical suspicion for HSV encephalitis despite initially non-supportive CSF findings. A previously described case involved a 49-year-old man who was admitted with a 2 h history of acute-onset aphasia and right hemiplegia [16]. After intracerebral hemorrhage was excluded, intravenous thrombolysis (IVT) was administered. Later, fluctuations in clinical symptoms were noted. The patient became increasingly somnolent, opening his eyes only after repeated verbal stimulation, and was unable to follow commands; motor aphasia reappeared. Over the subsequent 12 h, myoclonic seizures increased in frequency and became generalized on two occasions. His level of consciousness further deteriorated—he opened his eyes only in response to painful stimuli, and the only observed motor response was flexion of the right upper limb to pain. Viral encephalitis was suspected, and lumbar puncture was performed to obtain CSF for analysis. The CSF was clear but showed pleocytosis of 45 cells/mm^3^ (reference <5), along with an elevated protein concentration of 65 mg/dL. The diagnosis of HSV infection was confirmed by detection of viral DNA in the CSF using PCR. The patient was discharged in stable condition two days after completing antiviral therapy, although mild cognitive impairment persisted at the 3-month follow-up [16]. HSE can also mimic glioblastoma multiforme (GBM) in a patient previously diagnosed with this neoplasm [17]. The diagnosis may be challenging because HSE can clinically and radiologically mimic multicentric GBM. One reported case involved a 60-year-old man with glioblastoma treated with radiotherapy and temozolomide who developed HSV-1 encephalitis associated with profound neutropenia. Despite progressive neurological deterioration and a new temporal lobe lesion on MRI, CSF analysis showed no pleocytosis (0 WBC/mm^3^). HSV-1 infection was confirmed by CSF PCR, and viral reactivation was suspected. The patient subsequently died from multi-organ failure, highlighting that HSE should remain in the differential diagnosis of new neurological deterioration in GBM patients, even in the absence of CSF pleocytosis [17].

Elevated CSF protein concentration is a common finding in HSE, and therefore, the elevated protein level observed in our patient (113 mg/dL) should not be considered an atypical feature. Rather, the notable finding in this case was the absence of CSF pleocytosis despite elevated protein concentration and subsequently confirmed HSV-1 infection. Taken together, these reports demonstrate that HSE may present without CSF pleocytosis, including in both immunocompetent and immunocompromised patients (summarized in Table 1). However, the absence of pleocytosis should be interpreted in the context of the timing of lumbar puncture and the overall clinical presentation. In our patient, lumbar puncture was performed at least 8 days after the onset of the febrile illness. Therefore, the delayed timing of CSF sampling represents an important limitation, as we cannot determine whether the patient had previously developed a transient inflammatory CSF response that was no longer detectable at the time of lumbar puncture. Advanced age and the evolution of the disease may potentially influence the inflammatory response. Nevertheless, the absence of pleocytosis should not be considered sufficient to exclude HSE, particularly in patients with impaired consciousness and limited clinical assessment. An analysis of patients admitted to King Chulalongkorn Memorial Hospital between January 2002 and 2011 with PCR-confirmed or presumed viral CNS infections found that over the 10-year study period, 413 patients were diagnosed with viral neurological infections, with PCR-confirmed viral etiologies identified in 158 cases (38.3%). HSV was detected in the CSF of 43 patients (27.2%), including 23 cases of encephalitis and 20 cases of meningitis. Among the 23 patients with encephalitis, 6 (26.1%) had normal CSF white blood cell counts (<5 cells/mm^3^). One of these patients with normocellular CSF was HIV-positive [18]. The retrospective study of initial CSF profiles in 597 adult patients with all-cause encephalitis from two hospitals in Houston, Texas, and similarly in two hospitals in Baltimore, Maryland, revealed that among patients with infectious encephalitis, 47 out of 247 (19%) showed no pleocytosis, including 18 of 76 cases (23.7%) with HSV-1 encephalitis. Notably, the absence of pleocytosis was associated with a lower rate of acyclovir use, with treatment administered in 47.7% of patients without pleocytosis compared to 71.1% of those with pleocytosis (*p* < 0.001) [19]. An evaluation of all consecutive PCR-confirmed HSE cases treated at the Hospital of Ludwig Maximilian University in Munich, Germany, between January 1, 2013, and February 28, 2018, identified 18 patients, of whom 4 (22.2%) presented with normocellular CSF on admission [20].

The main limitation of this report is the single-case design, which limits the generalizability of the findings. Furthermore, the patient’s advanced age, pre-existing Alzheimer’s-type dementia, and markedly impaired level of consciousness limited the reliable assessment of memory, higher cortical functions, and other clinical features that might have supported the diagnosis of encephalitis. The lumbar puncture was performed at least 8 days after the onset of the febrile illness; therefore, the timing of CSF sampling may have influenced the absence of pleocytosis, and a transient earlier inflammatory response cannot be excluded. Nevertheless, the diagnosis of HSE was supported by the MRI abnormalities and detection of HSV-1 DNA in the CSF by PCR.

## 4. Conclusions

We report a case of HSE presenting with acute neurological symptoms despite normocellular CSF and the absence of laboratory markers of systemic inflammation during admission, including normal serum CRP and procalcitonin levels and no leukocytosis. HSE is a severe central nervous system disease associated with high mortality that requires rapid diagnosis and immediate initiation of antiviral therapy [15,21]. Although acyclovir was initiated promptly once MRI findings raised suspicion of encephalitis, this occurred after several days of clinical deterioration. In the presented case, early consideration of a viral etiology was crucial in a patient presenting with impaired consciousness, fever, and a history of previous infection with a herpes-group virus, despite the absence of typical inflammatory findings in the routine CSF examination. Diagnostic investigations, particularly brain MRI and CSF PCR testing for HSV, enabled confirmation of the diagnosis. This case highlights the importance of integrating the clinical presentation, neuroimaging, EEG findings, and molecular CSF diagnostics when evaluating suspected HSE, particularly when routine CSF analysis is non-inflammatory. The absence of CSF pleocytosis should not be used in isolation to exclude HSE, and the timing of CSF sampling should be considered when interpreting this finding. It also emphasizes the potential value of early brain MRI when the clinical history and neurological examination are limited. Rapid diagnosis and interdisciplinary management are crucial for early detection of HSE and timely initiation of appropriate treatment.

## Figures and Tables

**Table 1:** Selected published cases illustrating atypical cerebrospinal fluid findings and diagnostic challenges in herpes simplex encephalitis.

Reference	Sex/Age	Clinical Course	Imaging Findings	CSF Findings	HSE Confirmation	Outcome
[12]	M/72	HSE without CSF pleocytosis; two lumbar punctures	Temporal lobe hypodensity (edema) on CT	First LP: 1 WBC/μL, protein 0.45 g/L; second LP: 3 WBC/μL, protein 0.49 g/L	CSF HSV-1 PCR positive on the second lumbar puncture	Died
[13]	M/63	New-onset seizure; initially suspected autoimmune encephalitis; delayed fever	Initial MRI normal; subsequent HSE-compatible changes	4 WBC/μL, protein 42.7 mg/dL	HSV-1 PCR positive in CSF and serum	Discharged without sequelae
[14] (Case 1)	M/90	Fever, confusion; no focal deficits	CT normal	1 WBC/μL; protein 1.23 g/L	CSF HSV-1 PCR positive	Discharged
[14] (Case 2)	M/80	Seizure with fever; progression to status epilepticus	CT normal; EEG temporal slowing	1 WBC/μL; protein 0.78 g/L	CSF HSV-1 PCR positive	Died (day 5)
[16]	M/49	Stroke mimic; aphasia, hemiplegia; post-IVT seizures	CT negative for hemorrhage; subsequent MRI—cortical enhancement in the left frontal lobe on DWI	WBC 45 cells/mm^3^; protein 65 mg/dL	CSF HSV DNA PCR positive	Mild cognitive impairment at 3 months
[17]	M/60	GBM with neurological deterioration; HSE mimicking tumor progression	New temporal lobe lesion on MRI	0 WBC/mm^3^; protein 100 mg/dL	CSF HSV-1 PCR positive	Died (multi-organ failure)
Present case	F/85	Fever, progressive impairment of consciousness and generalized neurological deterioration; initially suspected ischemic stroke	Initial CT: left insular hypodensity; MRI: bilateral temporal and insular cortical diffusion restriction, more pronounced on the left	0 WBC/μL; protein 113 mg/dL	CSF HSV-1 PCR positive	Died (day 6 of hospitalization)

## Data Availability

All the data supporting the findings of this study are available upon request from the corresponding author.

## Abbreviations

The following abbreviations are used in this manuscript:

AMPA	α-Amino-3-Hydroxy-5-Methyl-4-Isoxazolepropionic Acid Receptor
CASPR2	Contactin-associated protein-like 2
CMV	Cytomegalovirus
CNS	Central Nervous System
CSF	Cerebrospinal Fluid
CT	Computed Tomography
DPPX	Dipeptidyl-Peptidase-Like Protein 6
DWI	Diffusion-Weighted Imaging
EEG	Electroencephalography
EV	Enterovirus
FLAIR	Fluid-Attenuated Inversion Recovery
GABA-B	Gamma-Aminobutyric Acid Type B Receptor
GBM	Glioblastoma Multiforme
HHV-6	Human Herpesvirus 6
HIV	Human Immunodeficiency Virus
HPeV	Human Parechovirus
HSE	Herpes Simplex Encephalitis
HSV	Herpes Simplex Virus
HSV-1	Herpes Simplex Virus Type 1
HSV-2	Herpes Simplex Virus Type 2
IgG	Immunoglobulin G
IVT	Intravenous Thrombolysis
LGI1	Leucine-Rich Glioma-Inactivated Protein 1
MRI	Magnetic Resonance Imaging
NMDA	N-Methyl-D-Aspartate Receptor
PCR	Polymerase Chain Reaction
VZV	Varicella-Zoster Virus
WBC	White Blood Cell
